# The study of metabolites from fermentation culture of *Alternaria oxytropis*

**DOI:** 10.1186/s12866-019-1408-8

**Published:** 2019-02-11

**Authors:** Runjie Song, Jinglong Wang, Lu Sun, Yajing Zhang, Zhenghui Ren, Baoyu Zhao, Hao Lu

**Affiliations:** 1 College of Veterinary Medicine, Northwest A&F University, Yangling, 712100 Shaanxi China; 2 Institute of Pratacultural Science, Tibet Academy of Agricultural and Animal Husbandry Sciences, Lhasa, 850000 Tibet China

**Keywords:** *Alternaria oxytropis*, Metabolites, Alkaloid, Endophytic fungus, Locoweeds

## Abstract

**Background:**

The indolizidine alkaloid-swainsonine is produced by an endophytic fungus *Alternaria oxytropis*, which was isolated from locoweeds. Swainsonine has many biological activities such as anti-tumorigenic, anti-viral and bacteriostatic. However, the full complement of metabolites produced *by Alternaria oxytropis* is not known. This study is a chemical analysis of *Alternaria oxytropis* metabolites, which not only unravels the potential compounds from the fermentation broth but also in which solvent are they extracted, facilitating industrial application.

**Results:**

*Alternaria oxytropis* isolated from *Oxytropis gansuensis* was cultured in Czapek’s medium for 30d to collect the fermentation broth. The fermentation broth is treated with methanol and then evaporated to dryness to obtain a concentrate of the fermentation broth. The concentrate is added with water for the subsequent fractional extraction with petroleum ether, chloroform, ethyl acetate and n-butanol. Different fractions of the extract were eluted by wet packing and dry loading. The obtained eluate was combined by TLC to detect the same fraction, and then characterized by GC-MS and LC-MS. The results of GC-MS showed that 105 different compounds existed in the petroleum ether, chloroform, and ethyl acetate phases of *Alternaria oxytropis* fermentation broth. Moreover, the results of LC-MS indicated that the fermentation broth of *Alternaria oxytropis* contained five alkaloids, 2-hydroxy-indolizidine, retronecine, lentiginosine, swainsonine and swainsonine N-oxide.

**Conclusions:**

In addition to swainsonine and swainsonine N-oxide, 2-hydroxy-indolizidine, retronecine and lentiginosine were identified as the secondary metabolites of *Alternaria oxytropis*. Other compounds were also detected including 5,6-dihydroergosterol, eburicol, lanosterol, and L-phenylalanyl-L-proline lactam, which have potential applications as drugs.

**Electronic supplementary material:**

The online version of this article (10.1186/s12866-019-1408-8) contains supplementary material, which is available to authorized users.

## Background

Locoweed is a generic term for toxic *Oxytropis* and *Astragalus* sp. plants that contain swainsonine. Swainsonine can cause neurological symptoms and pathological changes to animals that consume locoweeds. Locoweeds are the world’s leading poisonous plants and threaten livestock husbandry in the steppes [1, 2], which are widely distributed in the western provinces of China [3–7] and North America [8, 9].

Swainsonine is a water-soluble indolizidine alkaloid, which was originally extracted from *Swainsona canescens* [10]. In 1982, Molyneux et al. isolated swainsonine from *Astragalus lentiginosus*. Subsequently, Tulsiani et al. [11] demonstrated that swainsonine was the major toxic component of locoweeds. Swainsonine can inhibit the activity of lysosomal α-mannosidase and Golgi mannosidase II in mammalian cells and cause the disorder of intracellular oligosaccharides metabolism and glycoprotein synthesis, leading to cell vacuolar degeneration and tissue and organ function disorder [10–12]. Initially, the researchers thought that swainsonine was a metabolite of locoweed, but upon further study, the toxicity of locoweed was associated with a slow-growing endophytic fungi, *Alternaria oxytropis* [13–16], and *Alternaria oxytropis* was demonstrated to produce swainsonine. Swainsonine causes beneficial pharmacological effects such as antiviral, bacteriostatic and anti-tumorigenic [17–19], in addition to inhibiting tumor cell metastasis and growth [20]. Muchmore et al. [21] found that swainsonine can enhance the killing activity of TNF-a on tumor cells and enhance the killing activity of human monocytes on tumor cells cultured in vitro. Subsequently, You et al. [22] found that swainsonine can directly inhibit the growth of liver cancer cells and enhance the response of liver cancer cells to paclitaxel. Swainsonine also enhances the immune system [23–26]. Scott et al. showed that the susceptibility of apolipoprotein E knockout mice to cardiovascular disease is related to the sensitivity of mice to immunomodulatory effects of swainsonine [27].

The limited sources of swainsonine, difficulties inartificial synthesis, the low extraction efficiency, and high market price have limited the development of swainsonine for anti-cancer and anti-tumor applications. At present, there are three main sources of swainsonine [28]. The first is chemical synthesis. Due to the presence of four chiral carbon atoms in the chemical structure of swainsonine, a large amount of the chiral isomer of swainsonine is produced during the artificial synthesis and separation is very difficult [29]. The second is extraction from plants. The extensive extraction of swainsonine from plants can cause irreparable damage to grassland pastures and the extraction process is very complicated [30]. The third method is extraction from fungal fermentation. Biological fermentation has some unique characteristics, such as low cost, easy to control fermentation conditions, and high yields [31, 32].

Endophytic fungi of plants produce rich and diverse metabolites with a wide range of active substances. Therefore, the potential for the separation of new active compounds from them is very large and has drawn considerable attention as a potential source of prodrugs [33, 34]. For the analyses of the chemical composition of fungal metabolites, it is common to concentrate the fungal fermentation broth to produce an extract and then disperse the extract with distilled water, followed by highly polar to nonpolar solvents in a fractional extraction [35, 36]. Zhang [37] found that fermentation broth of *Alternaria oxytropis* has an obvious inhibitory effect on bacteria, with the strongest antibacterial activity from ethyl acetate extracts (*E.coli* antibacterial rate as high as 89.6%). However, it was not clear what compounds were present*.*

In this study, we perform a chemical screening from petroleum ether, chloroform and ethyl acetate phase of the fractional extraction, which not only unravels the potential compounds from the fermentation broth but also in which solvent are they extracted, facilitating industrial application.

## Results

### Fractional extraction of the broth followed by chemical analysis of the solvents

To detect the relatively small polar and volatile substances in the metabolites of *Alternaria oxytropis*, we analyzed the petroleum ether phase, chloroform phase, and ethyl acetate phase by GC-MS. First, we dispersed concentrates of the metabolites of *Alternaria oxytropis* by adding water, and then extracted it with petroleum ether according to the polarity of the extractant from low to high (petroleum ether < chloroform < ethyl acetate < n-butanol).

### GC-MS analysis of petroleum ether phase in metabolites of *Alternaria oxytropis*

**Fig. 1:**
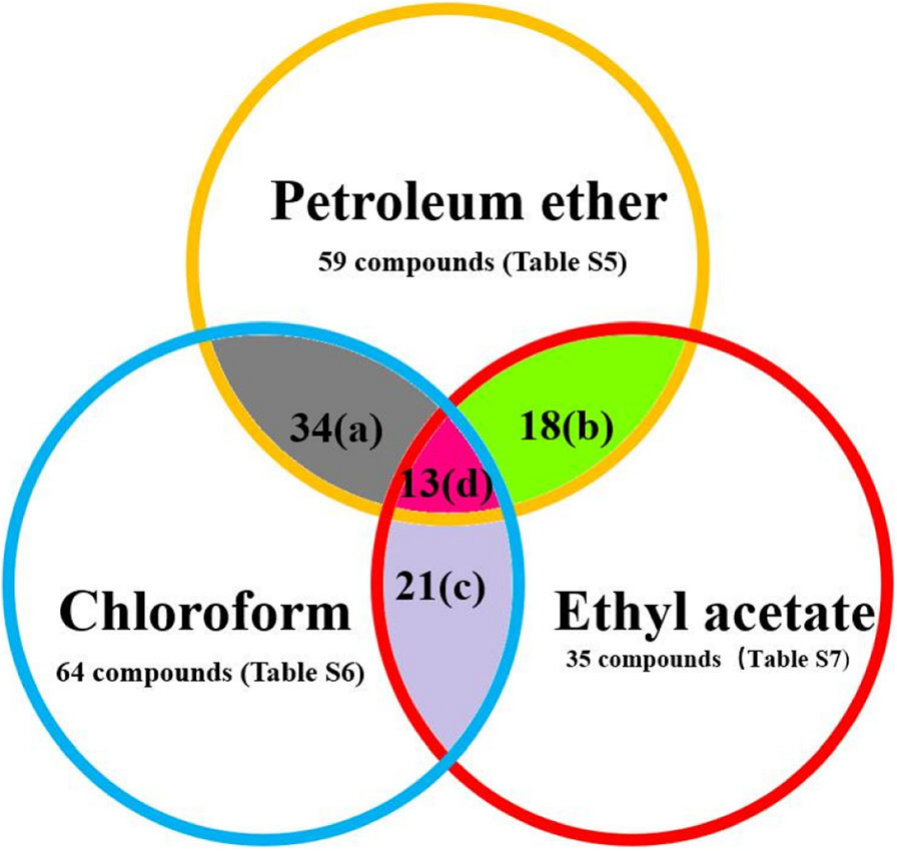
Summary of chemical substances in *Alternaria oxytropis* fermentation broth. **a** 34 identical compounds isolated from the petroleum ether phase and chloroform phase of *Alternaria oxytropis* (Additional file 1: Table S1)*.* **b** 18 identical compounds isolated from petroleum ether phase and ethyl acetate phase of *Alternaria oxytropis* (Additional file 1: Table S2). **c** 21 identical compounds isolated from chloroform phase and ethyl acetate phase of *Alternaria oxytropis* (Additional file 1: Table S3). **d** 13 identical compounds isolated from three phases of *Alternaria oxytropis* (Additional file 1: Table S4)

After that, 3 g of total petroleum ether phase extract was eluted and purified into S-1, S-2, and S-3 fractions, and the purified sample was dissolved in methanol for GC-MS analysis. According to GC-MS data, 42, 16 and 15 monomer compounds were identified in S-1, S-2 and S-3 fractions of the petroleum ether phase, respectively. The petroleum ether phase of *Alternaria oxytropis* metabolites contained a total of 59 compounds with the exception of repeats (Fig. 1 and Additional file 1: Tables S1 and S2), including (22E)-Ergosta-5,7,9(11),22-teeraen-3,β-ol, 5,6-dihydroergosterol, eburicol, and lanosterol. The total ion chromatogram and compound identification results are presented in Additional file 1: Table S5 and Additional file 2: Figures S1–S3.

### GC-MS analysis of chloroform phase in metabolites of *Alternaria oxytropis*

After extraction with petroleum ether, the concentrates of *Alternaria oxytropis* metabolites were extracted with more polar chloroform. Three grams of total petroleum ether phase extract were eluted and purified into L-1, L-2, and L-3 fractions, and the purified sample was dissolved in methanol for GC-MS analysis. After that, the metabolites of *Alternaria oxytropis* were detected by GC-MS and identified by comparison with the MS database. Chloroform phase L-1, L-2, L-3 were identified as 40, 15, 18 monomer compounds respectively. After repeats were removed (Fig. 1 and Additional file 1: Table S3), the fermentation broth of the chloroform phase contained 64 compounds, including L-phenylalanyl-L-proline lactam. The total ion chromatogram and compound identification results are shown in Additional file 1: Table S6 and Additional file 2: Figures S4–S6.

### GC-MS analysis of ethyl acetate phase in metabolites of *Alternaria oxytropis*

After the concentrates of the metabolites of *Alternaria oxytropis* were extracted with petroleum ether and chloroform, most of the small polar substances had been extracted, so the extraction was carried out with the more polar ethyl acetate. Five grams of the total ethyl acetate phase extract were eluted and purified into Y-1, Y-2, and Y-3 fractions, which were analyzed by GC-MS and aligned in the MS database. The results showed that 26, 12 and 10 monomer compounds were identified in the ethyl acetate phase samples Y-1, Y-2 and Y-3, respectively, and some of them were duplicated (Fig. 1 and Additional file 1: Table S4). Therefore, the metabolites of *Alternaria oxytropis* finally obtained in the ethyl acetate phase contained a total of 35 compounds, including 5,6-dihydroergosterol, eburicol. The total ion chromatogram and compound identification results are as follows (Additional file 1: Table S7 and Additional file 2: Figures S7–S9).

### LC-MS analysis on JZ1–4 of alkaline n-butanol phase in metabolites of *Alternaria oxytropis*

After extraction with the above three extractants, most of the small polar volatile compounds have been detected by GC-MS. However, highly polar and non-volatile compounds were not likely to have been completely extracted by the above three extractants and some of the less volatile substances may not have been completely detected by GC-MS. Therefore, it was necessary to extract with a more polar n-butanol extractant, followed by LC-MS. Four grams of the fermentation broth from the alkaline n-butanol phase extract were eluted and purified into JZ1, JZ2, JZ3 segments, and the purified material was processed for LC-MS analysis. Four alkaloids (2-hydroxy-indolizidine, retronecine, lentiginosine and swainsonine) were identified in the alkaline n-butanol phase of *Alternaria oxytropis* metabolites, JZ1, JZ2 and JZ3, respectively (Table 1 and Additional file 2: Figures S10–S13). JZ4 preliminary identified five kinds of alkaloids. In addition to the above 4 species, swainsonine N-oxide was also detected.

**Table 1:**
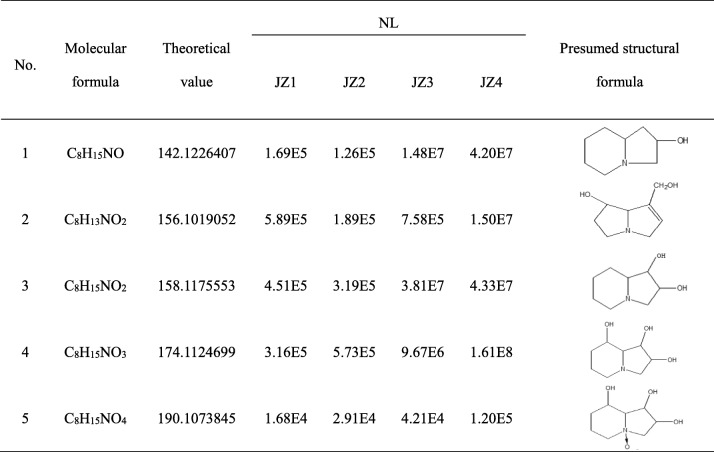
LC-MS analysis of JZ1–4 segment of *Alternaria oxytropis*

### LC-MS analysis on JZ5 of alkaline n-butanol phase acetylation in metabolites of *Alternaria oxytropis*

To further verify the presence of 2-hydroxy-indolizidine, retronecine, lentiginosine, swainsonine, and swainsonine N-oxide, the alkaline n-butanol phase of *Alternaria oxytropis* fermentation broth was acetylated again for LC-MS detection. The alkaline n-butanol phase of the acetylated *Alternaria oxytropis* metabolites initially identified eight compounds and four further species that could not be completely determined due to the slightly lower signal strength compared to the error tolerance (Table 2, Additional file 2: Figures S14–S16). Compared with the pre-acetylation LC-MS analysis, these components migrated ahead of the five alkaloids obtained after varying degrees of acetylation. Among them, No. 2, 3 was first and second acetylation of retronecine, No. 4, 5 was first and second acetylation of lentiginosine, No. 7, 8 was second and third acetylation of swainsonine, and No. 10, 11 was second and third acetylation of swainsonine N-oxide, respectively. This result further supported the detection of 2-hydroxy-indolizidine, retronecine, lentiginosine, swainsonine, and swainsonine N-oxide in the n-butanol phase of the *Alternaria oxytropis* fermentation broth.

**Table 2:** LC-MS analysis of JZ5 segment of *Alternaria oxytropis*

No.	Molecular formula	Theoretical value	NL
1	C_10_H_17_NO_2_ = 183	184.1332053	7.55E4
2	C_10_H_15_NO_3_ = 197	198.1124699	1.87E5
3	C_12_H_17_NO_4_ = 239	240.1230345	9.21E5
4	C_10_H_17_NO_3_ = 199	200.1281199	1.07 E5
5	C_12_H_19_NO_4_ = 241	242.1386846	7.56 E6
6	C_10_H_17_NO_4_ = 215	216.1230345	5.35E4
7	C_12_H_19_NO_5_ = 257	258.1335992	3.47E5
8	C_14_H_21_NO_6_ = 299	300.1441639	1.19E8
9	C_10_H_17_NO_5_ = 231	232.1179491	3.32E4
10	C_12_H_19_NO_6_ = 273	274.1285138	1.56E5
11	C_14_H_21_NO_7_ = 315	316.1390785	1.93E6
12	C_16_H_23_NO_8_ = 357	358.1496431	4.00E4

Note: maximum signal strength greater than 1.00E5 is the allowable range of error

## Discussion

The bioactive metabolites produced by endophytic microorganisms are potential sources of novel biomolecules that may have applications in medicine and bioindustry [38]. Many studies have focused on the indolizidine alkaloid swainsonine, a secondary metabolite of the endophytic fungus *Alternaria oxytropis* [39]. However, there are few reports on the primary metabolites of the endophytic fungus *Alternaria oxytropis.* In our study, the petroleum ether, chloroform and ethyl acetate phase of *Alternaria oxytropis* metabolites were analyzed by GC-MS, and 59, 64, 35 kinds of monomer compounds were identified, respectively, by comparison with the mass spectrometry database. Compounds identified were alkanes and esters, and included alcohols, aldehydes, organic acids and nitrogen-containing compounds. The compounds 5,6-dihydroergosterol, eburicol, lanosterol, and L-phenylalanyl-L-proline lactam have good medicinal potential. For the n-butanol phase, we used a high-resolution LC-MS combination technique to analyze the swainsonine and its polar and non-volatile materials. The compounds 2-hydroxy-indolizidine, retronecine, lentiginosine, swainsonine, and swainsonine N-oxide were detected from the n-butanol phase of *Alternaria oxytropis* metabolites by LC-MS.

We identified steroid substances in the petroleum ether phase and the ethyl acetate phase, mainly sterols, sterols due to their solid state, also known as sterols, cyclopentane perhydrophenanthrene skeleton as an alcohol compound divided into phytosterols, animal sterols and steroid sterols. Zhang [37] confirmed that the ethyl acetate fraction from fermentation broth of *Alternaria oxytropis* had the strongest antibacterial activity (up to 89.6% inhibition against *E. coli*). The antimicrobial substance has not been reported yet. Herath et al. [40] found that the ethyl acetate extracts of *Armillaria tabescens* showed antimicrobial activity against the fungi *Candida albicans*, *Cryptococcus neoformans* and the bacteria *Escherichia coli* and *Mycobacterium intracellulare*. The ergosterol detected in this study is mainly found in fungi. Li et al. [5] obtained ergosterol and another steroid from alcohol and water extracts of the mycelium of *Hericium erinaceum*. Sterols can enhance the body’s resistance to disease and have significant antibacterial and antitumor activity [41], serve not only as a precursor to the production of vitamin D2, but also produce hormonal drugs such as cortisone and hormone progesterone. Zheng et al. [42] also detected ergosterol from secondary metabolites of the ginseng endophytic fungus *Penicillium melinii* Yuan-25 and *Penicillium janthinellum* Yuan-27. In addition, Kang et al. [43] extracted Chaga mushrooms with different solvents and found that the active ingredient ergosterol peroxide inhibited the proliferation of CRC cell lines and effectively inhibited colitis-associated colon cancer in AOM/DSS-treated mice. Therefore, the inhibitory substances in the ethyl acetate section in metabolites of *Alternaria oxytropis* may be steroidal substances. Secondly, this test detected the presence of lanosterol from the petroleum ether phase, which also has its own specific use. In 2015, the researchers found that lanosterol can effectively reduce the possibility of protein agglomeration in human lens cells, thereby reducing the chance of cataract formation. Moreover, the use of lanosterol in dogs and rabbits with cataracts showed that they reduced their cataract symptoms and a clearer field of vision, and currently the primary method of treatment for cataracts is surgical treatment [44]. Therefore, researchers concluded that lanosterol eye drops may become a novel strategy in prevention and treatment of cataract [44]. In this study, the percentage of lanosterol detected in the petroleum ether phase of the *Alternaria oxytropis* was 2.76%, so that the *Alternaria oxytropis* can be used to obtain the substance for treatment of cataract diseases.

In addition to steroidal substances, cyclic dipeptides (CDPs) are found in many endogenous fungal secondary metabolites. Tao et al. [45] and Lyu et al. [46] identified a variety of cyclic dipeptide compounds in the metabolites of the endophytic fungi *Phomopsis sp*. ZZF08 and *Trichoderma harzianum*, respectively. The cyclic dipeptide (CDPs) compounds beyond the steroidal substances have attracted the attention of a large number of medical workers due to their varying degrees of anti-tumor activity, particularly, the cyclic (phenylpropionate) dipeptide existed in the ethyl acetate phase of *Alternaria oxytropis* has been found that the inhibitory rate of the growth of cancer cells is 50% at a concentration of 10 μg mL^− 1^ and the inhibitory effect on the growth of colon cancer cells, breast cancer cells, cervical cancer cells and significant inhibitory ability, it is a promising anti-tumor drugs. In addition, 23 species of low molecular weight cyclopeptidyl peptides with certain insecticidal activities were isolated from *Metarhizium anisopliae* [47]. Xu et al. [48] considered that natural active cyclic peptides such as cyclosporidium A, A, CP, AST, etc. have a good medicinal value. As can be seen, it is necessary for us to further study the cyclopeptides from *Alternaria oxytropis*.

Alkaloids such as indolizidine [49], pyrrolizidine, and quinolizidine [50] have been the focus of medical and pharmacological research. Alkaloids, as a class of active nitrogenous basic organic compounds ubiquitous in nature, are not only the most important active ingredients in Chinese herbal medicine such as Ariston sulfate [51], which have anti-retroviral activity and anti-tumor activity, but also the secondary metabolites of many kinds of microorganisms. Therefore, for many years, domestic and foreign scholars have been looking for alkaloids, which are beneficial to human health from different sources such as plants and microorganisms. We detected 2-hydroxy-indolizidine, retronecine, lentiginosine, swainsonine, and swainsonine N-oxide from the n-butanol phase of the metabolites of *Alternaria oxytropis*. Swainsonine has a significant anticancer and antitumor effect. It not only can directly inhibit tumor cells and change the surface glycosylation of tumor cells, but also can promote the killing effect of cytokines or chemotherapeutics on tumor cells and exert anti-tumor effect by inducing apoptosis of tumor cells. Additionally, it has been shown that swainsonine N-oxide is as effective as swainsonine in inhibiting alpha-mannosidase [﻿1﻿]. To better define the swainsonine N-oxide, Gardner et al. [52] established an effective method for the detection of swainsonine N-oxide and detected swainsonine N-oxide fro*m Alternaria oxytropis.* Beyond swainsonine, and swainsonine N-oxide, the other three alkaloids were isolated and identified from the metabolites of *Alternaria oxytropis*. This is similar to the previous study on alkaloid detection in locoweed plants [53]. These swainsonine analogues may be involved in the metabolic pathway of swainsonine, providing a theoretical basis for further studies on swainsonine derived from a fungal endophyte, *Alternaria oxytropis.*

## Conclusion

In conclusion, some compounds as potential drugs, like 5,6-dihydroergosterol, eburicol, lanosterol, L-phenylalanyl-L-proline lactam were detected by GC-MS from metabolites of *Alternaria oxytropis*. Moreover, in addition to swainsonine and swainsonine N-oxide, 2-hydroxy-indolizidine, retronecine, lentiginosine were firstly identified by LC-MS from metabolites of *Alternaria oxytropis.* The above results will provide the basis for the future research on the pharmacological activity and industrial application of the metabolites of *Alternaria oxytropis.*

## Materials and methods

### Experiment material

The culture of *Alternaria oxytropis* was provided by the Laboratory of Toxicology, College of Veterinary Medicine, Northwest A&F University. Swainsonine, swainsonine N-oxide, 2-hydroxy-indolizidine, retronecine, lentiginosine and standards were provided by the Chengdu Must Bio-Technology Co., Ltd. (Assay by HPLC ≥98%).

### Fermentation culture of *Alternaria oxytropis*

The strain of *Alternaria oxytropis* was inoculated into PDA medium by using the “tip-picking method” and cultured at 25 °C for 7 days. Then the 5 mm strain was transferred to Czapek’s broth for fermentation culture at room temperature (25 °C), 150 r min^− 1^, for 30 d. After fermentation, the fermentation broth was first separated by using an 8-layer miracloth, and then the fermentation broth is filtered again using a 0.22 μm filter. After filtration, a total of 12 L fermentation broth was obtained; the mycelium was dried at 45 °C and then ground to a powder in a mortar and stored at 4 °C for later use.

### Isolation and extraction of metabolites of *Alternaria oxytropis*

**Fig. 2:**
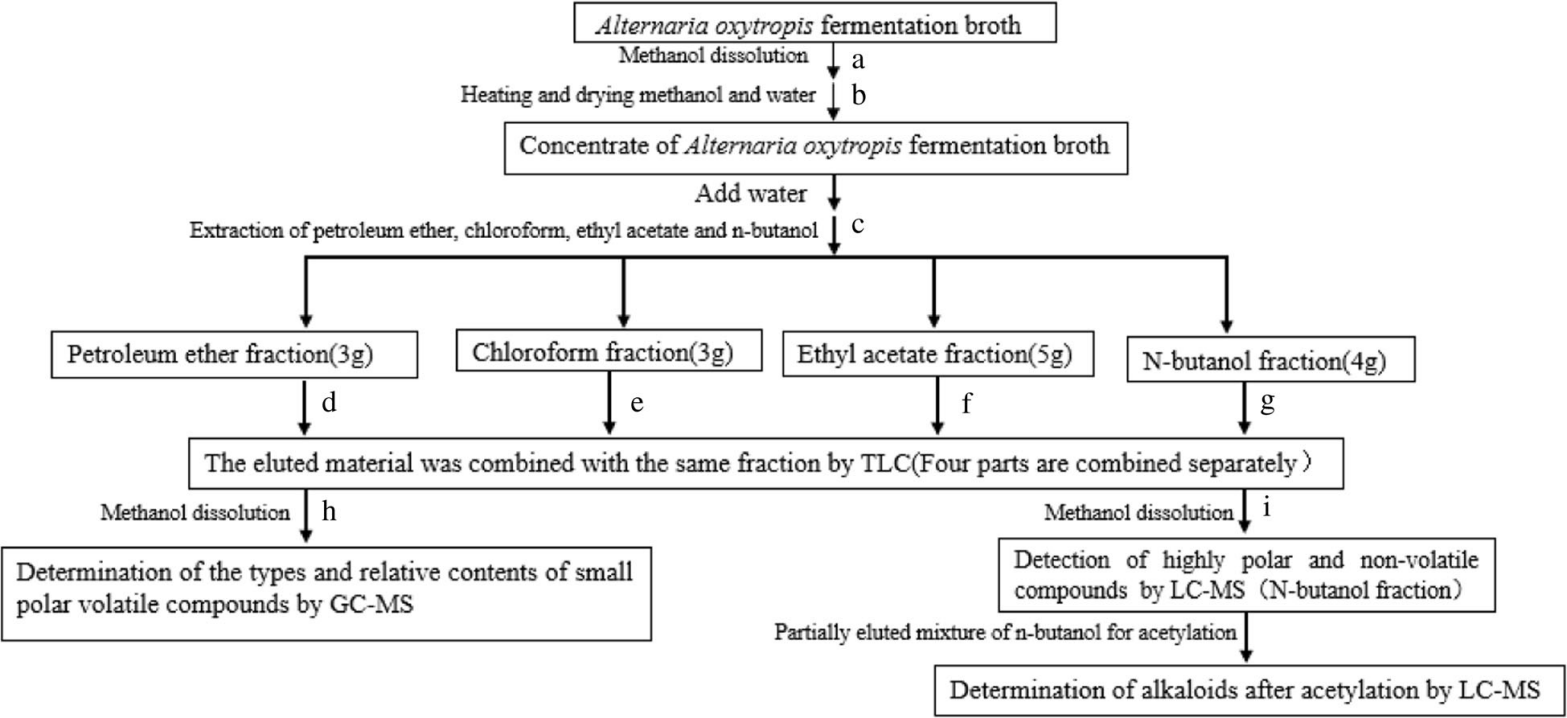
Flowchart of chemical composition analysis of metabolites of *Alternaria oxytropis.* **a** The 12 L total fermentation broth were dissolved with appropriate amount of methanol (V = 1:1) overnight at 4 °C, the 23 L supernatant was filtered three times after addition of the alcohol extract. **b** The methanol and water in the supernatant are volatilized to obtain concentrated substances of *Alternaria oxytropis* fermentation broth. **c** The 150 g concentrate is added with water for the subsequent extraction of petroleum ether, chloroform, ethyl acetate and n-butanol. **d** Packing the column after mixing ten times the weight of the petroleum ether extract with petroleum ether; The petroleum ether phase extract is dissolved in methanol and mixed with the same quality of silica to form a powder, and then subjected to gradient elution with petroleum ether, petroleum ether: acetone (V:V = 7:3, 6:4). **e** The silica is mixed with chloroform and added to the column with 10 times the weight of the petroleum ether extract; the chloroform phase extract is dissolved in methanol and mixed with the same quality of silica to form a powder, and then subjected to gradient elution with chloroform, chloroform: acetone (V:V = 5:5, 10:13). **f** The method is the same as e, using chloroform: methanol: aqueous ammonia: water (V:V:V:V = 70:26:2:2) for elution. **g** The method is the same as e, chloroform: methanol: ammonia: water (9:1:0.1:0.1,8:1:0.1:0.1, 7:2:0.1:0.1, 70:26:2:2) for elution. **h** and **i** The purified samples were dissolved in methanol for GC-MS and LC-MS detection. The three independent experiments are presented

The 12 L total fermentation broth were dissolved with appropriate amount of methanol (V = 1:1) overnight at 4 °C, the 23 L supernatant was filtered three times after addition of the alcohol extract. The methanol and water in the supernatant are volatilized to obtain concentrated substances of *Alternaria oxytropis* fermentation broth. The 150 g concentrate is added with water for the subsequent fractional extraction with petroleum ether, chloroform, ethyl acetate and n-butanol. The detailed flowchart is shown in Fig. 2.

### GC-MS analysis of petroleum ether phase in metabolites of *Alternaria oxytropis*

The 3 g total petroleum ether extract was dissolved in petroleum ether and mixed with 3 g of silica gel, using wet packing and dry loading. Followed by a gradient of petroleum ether, petroleum ether: acetone (V:V = 7:3, 6:4) collected a total of 150 copies for each 10 mL. The eluate thus obtained was analyzed by TLC and the same fractions were combined. The 1–60, 61–86 and 87–150 fractions were combined into S-1, S-2 and S-3. The purified sample was dissolved in methanol, and the type and relative content of alkaloids were detected by GC-MS (Shimadzu, Japan). The chromatographic column ZB-5MSI 5% Phenyl-95% DiMethylpolysiloxane(30 m × 0.25 mm × 0.25 μm)fused silica capillary column (Chromatographic Technology Research and Development Center, Lanzhou Institute of Chemical Physics, Chinese Academy of Sciences). Column temperature 50 °C (reserved 2 min), 5 °C min^− 1^ heating to 310 °C, for 10 min; running time: 64 min; vaporization chamber temperature 250 °C; the carrier gas was He (99.999%); the column pressure was 7.65 psi, the carrier gas flow rate was 1.0 mL min^− 1^, splitless, and the solvent delay time was 3.0 min. Mass spectrometry conditions: ion source for the EI source; Ion source temperature 230 °C; quadrupole temperature 150 °C; electron energy 70 eV; emission current 34.6 μA; multiplier voltage 1294 V; interface temperature 280 °C; mass range 29–500 amu.

### GC-MS analysis of chloroform phase in metabolites of *Alternaria oxytropis*

The 3 g total chloroform phase extract was dissolved in methanol, mixed with 3 g silica gel (200 mesh), using wet packing, dry loading. Followed by a gradient elution with chloroform, chloroform: acetone (V:V = 5:5, 10:13), collected a total of 200 copies for each 10 mL. The eluate thus obtained was analyzed for the same fractions by TLC, that is, fractions 1–45, 46–56 and 57–200 were combined into L-1, L-2 and L-3.

### GC-MS analysis of ethyl acetate phase in metabolites of *Alternaria oxytropis*

Dissolve 5 g total ethyl acetate extract in methanol, mix with 5 g silica gel (200 mesh), and use wet packing method to dry the sample. Elution was carried out with chloroform: methanol: aqueous ammonia: water (V:V:V:V = 70:26:2:2). The resulting eluate was combined by the TLC test to the same fraction. The fractions from 1 to 24, 25–70 and 71–216 were combined into the Y-1, Y-2 and Y-3.

### LC-MS analysis of alkaline n-butanol in metabolites of *Alternaria oxytropis*

The 4 g alkaline n-butanol phase extract was dissolved in methanol, mixed with 4 g silica gel (200 mesh), using wet column packing and dry loading. A total of 55 fractions collected by each 20 mL were sequentially eluted with a gradient of chloroform: methanol: ammonia: water (9:1:0.1:0.1,8:1:0.1:0.1, 7:2:0.1:0.1, 70:26:2:2). The eluates thus obtained were combined by the TLC test with the same fractions. The fractions 1–3, 4–9, 10–22 and 23–55 were combined into JZ-1, JZ-2, JZ-3 and JZ-4, respectively. Take appropriate column chromatography JZ1, JZ2, JZ3, JZ4 segment mixture in a triangular flask, followed by dissolved in anhydrous pyridine and add an equal volume of acetic anhydride (Sinopharm) at room temperature for 24 h and stirring. After the reaction was added deionized water, CHCl_3_ extracted several times after the merger. CHCl_3_ was recovered to give residue JZ-5. The JZ1, JZ2, JZ3, JZ4 and acetylated JZ5 were dissolved, centrifuged and filtered with 1 mL of methanol. Diluted with 50% MeOH & 0.1% FA solvent, 10,000 times JZ1–4 samples and 2000 times JZ5 samples were analyzed by LC-MS. The detection conditions were as follows: mobile phase: A: 0.1% FA (formic acid) H_2_O B: 0.1% FA CAN (acetonitrile); chromatographic column: Agilent Poreshell EC-120, 3.0 × 100 mm 2.7 Micron; flow rate: 0.3 L min^− 1^; sample volume: 5 L; mass spectrometry using positive ion mode a full scan; scan range: 100–400 m z^− 1^.

## Additional files

Additional file 1: **Tables S1-S7.** Compounds isolated from petroleum ether, chloroform, ethyl acetate and n-butanol phase of *Alternaria oxytropis*. (DOC 223 kb)

Additional file 2: **Figures S1-S16.** The GC-MS and LC-MS analysis of petroleum ether, chloroform, ethyl acetate and n-butanol phase in metabolites of *Alternaria oxytropis*. (DOCX 1545 kb)
