# Supplementary material for: The study of metabolites from fermentation culture of Alternaria oxytropis
Source: BMC Microbiol. 2019 Feb 11;19:35. doi: 10.1186/s12866-019-1408-8 (PMC6369557; doi:10.1186/s12866-019-1408-8)
Supplement: Supplementary file 1 — Tables S1-S7. Compounds isolated from petroleum ether, chloroform, ethyl acetate and n-butanol phase of Alternaria oxytropis. (DOC 223 kb) [file 12866_2019_1408_MOESM1_ESM.doc]

Table 1 Same compounds isolated from petroleum ether phase and chloroform phase of *Alternaria oxytropis*

| Type | Compound | MF | MW |
| --- | --- | --- | --- |
| Alkanes  Olefin  Acids  Esters  Nitrogenous compounds | 1,1’-Oxybis-Dodecane  2-Methyl-Tricosane  Docosane  Eicosane  Hentriacontane  Heptacosane  Heptadecane  Hexacosane  Nonacosane  Nonadecane  Octacosane  Octadecane  Pentacosane  Triacontane  Tricosane  Tetracosane  Squalene  Linoleic acid  Oleic acid  Butyl phthalate  Dioctyl phthalate  Ethyl oleate  Isobutyl phthalate  Isooctyl phthalate  Methyl behenate  Methyl lignocerate  Methyl linoleate  Methyl oleate  Methyl palmitoleate  Methyl stearate  palmityl palmitate  1,4-diaza-2,5-dioxo-3-isobutyl bicyclo [4,3,0] nonane  3-benzyl-1,4-diaza-2,5-dioxobicyclo [4,3,0] nonane  Methyl N-（phenylacetyl）glycine | C_24_H_50_O  C_24_H_50_  C_22_H_46_  C_20_H_42_  C_31_H_64_  C_27_H_56_  C_17_H_36_  C_26_H_54_  C_29_H_60_  C_19_H_40_  C_28_H_58_  C_18_H_38_  C_25_H_52_  C_30_H_62_  C_23_H_48_  C_24_H_50_  C_30_H_50_  C_18_H_32_O_2_  C_18_H_34_O_2_  C_16_H_22_O_4_  C_24_H_38_O_4_  C_20_H_38_O_2_  C_16_H_22_O_4_  C_24_H_38_O_4_  C_23_H_46_O_2_  C_25_H_50_O_2_  C_19_H_34_O_2_  C_19_H_36_O_2_  C_17_H_32_O_2_  C_19_H_38_O_2_  C_32_H_64_O_2_  C_11_H_18_N_2_O_2_  C_14_H_16_N_2_O_2_  C_11_H_13_NO_3_ | 354  338  310  282  436  380  240  366  408  268  394  254  352  422  324  338  410  280  282  278  390  310  278  390  354  382  294  296  268  298  480  210  244  207 |

Table 2 Same compounds isolated from petroleum ether phase and ethyl acetate phase of *Alternaria oxytropis*

| Type | Compound | MF | MW |
| --- | --- | --- | --- |
| Alkanes  Olefin  Alcohols  Acids  Esters  Nitrogenous compounds | Heptacosane  Nonacosane  Octacosane  Squalene  5,6-dihydroergosterol  Eburicol  Linoleic acid  Dioctyl phthalate  Isooctyl phthalate  Methyl cerotate  Methyl lignocerate  Methyl linoleate  Methyl oleate  Methyl stearate  Methyl tricosanoate  1,4-diaza-2,5-dioxo-3-isobutyl bicyclo [4,3,0] nonane  Methyl N-（phenylacetyl）glycine  Erucamide | C_27_H_56_  C_29_H_60_  C_28_H_58_  C_30_H_50_  C_28_H_46_O  C_31_H_52_O  C_18_H_32_O_2_  C_24_H_38_O_4_  C_24_H_38_O_4_  C_27_H_54_O_2_  C_25_H_50_O_2_  C_19_H_34_O_2_  C_19_H_36_O_2_  C_19_H_38_O_2_  C_24_H_48_O_2_  C_11_H_18_N_2_O_2_  C_11_H_13_NO_3_  C_22_H_43_NO | 380  408  394  410  398  440  280  390  390  410  382  294  296  298  368  210  207  337 |

Table 3 Same compounds isolated from chloroform phase and ethyl acetate phase of *Alternaria oxytropis*

| Type | Compound | MF | MW |
| --- | --- | --- | --- |
| Alkanes  Olefin  Aldehydes  Ketone  Acids  Esters  Nitrogenous compounds | Heptacosane  Nonacosane  Octacosane  Squalene  4-(dimethylamino)-Benzaldehyde  5-Oxymethylfurfurole  2,3-dihydro-3,5-dihydroxy-6-methyl-4H-Pyran-4-one  Benzeneacetic acid  Linoleic acid  Dioctyl phthalate  Isooctyl phthalate  Methyl lignocerate  Methyl linoleate  Methyl oleate  Methyl Palmitate  Methyl stearate  Monomethyl succinate  1,4-diaza-2,5-dioxo-3-isobutyl bicyclo [4,3,0] nonane  1,4-diaza-2,5-dioxobicyclo [4,3,0] nonane  Methyl N-(phenylacetyl) glycine  Methyl pyroglutamate | C_27_H_56_  C_29_H_60_  C_28_H_58_  C_30_H_50_  C_9_H_11_NO  C_6_H_6_O_3_  C_6_H_8_O_4_  C_8_H_8_O_2_  C_18_H_32_O_2_  C_24_H_38_O_4_  C_24_H_38_O_4_  C_25_H_50_O_2_  C_19_H_34_O_2_ C_19_H_36_O_2_  C_17_H_34_O_2_  C_19_H_38_O_2_  C_5_H_8_O_4_  C_11_H_18_N_2_O_2_  C_7_H_10_N_2_O_2_  C_11_H_13_NO_3_  C_6_H_9_NO_3_ | 380  408  394  410  149  126  144  136  280  390  390  382  294  296  270  298  132  210  154  207  143 |

Table 4 Same compounds isolated from three phases of *Alternaria oxytropis*

| Type | Compound | MF | MW |
| --- | --- | --- | --- |
| Alkanes  Olefin  Acids  Esters  Nitrogenous compounds | Heptacosane  Nonacosane  Octacosane  Squalene  Linoleic acid  Dioctyl phthalate  Isooctyl phthalate  Methyl linoleate  Methyl oleate  Methyl palmitate  Methyl stearate  1,4-diaza-2,5-dioxo-3-isobutyl bicyclo [4,3,0] nonane  Methyl N-(phenylacetyl) glycine | C_27_H_56_  C_29_H_60_  C_28_H_58_  C_30_H_50_  C_18_H_32_O_2_  C_24_H_38_O_4_  C_24_H_38_O_4_ C_19_H_34_O_2_  C_19_H_36_O_2_ C_17_H_34_O_2_ C_19_H_38_O_2_ C_11_H_18_N_2_O_2_  C_11_H_13_NO_3_ | 380  408  394  410  280  390  390  294  296  270  298  210  207 |

Table 5 Compounds isolated from petroleum ether phase of *Alternaria oxytropis*

| Type | Compound | MF | MW |
| --- | --- | --- | --- |
| Alkanes | 1,1’-Oxybis-Dodecane | C_24_H_50_O | 354 |
|  | 1-Cyclopentyl-Heneicosane | C_26_H_52_ | 364 |
|  | 2-Methyl-Tricosane | C_24_H_50_ | 338 |
|  | Cycloeicosane | C_20_H_40_ | 280 |
|  | Docosane | C_22_H_46_ | 310 |
|  | Eicosane | C_20_H_42_ | 282 |
|  | Hentriacontane | C_31_H_64_ | 436 |
|  | Heptacosane | C_27_H_56_ | 380 |
|  | Heptadecane | C_17_H_36_ | 240 |
|  | Hexacosane | C_26_H_54_ | 366 |
|  | Nonacosane | C_29_H_60_ | 408 |
|  | Nonadecane | C_19_H_40_ | 268 |
|  | Octacosane | C_28_H_58_ | 394 |
|  | Octadecane | C_18_H_38_ | 254 |
|  | Pentacosane | C_25_H_52_ | 352 |
|  | Tetracosane | C_24_H_50_ | 338 |
|  | Triacontane | C_30_H_62_ | 422 |
|  | Tricosane | C_23_H_48_ | 324 |
| Olefin | 1-Docosene | C_22_H_44_ | 308 |
|  | 1-Hexacosene | C_26_H_52_ | 364 |
|  | 1-Octadecene | C_18_H_36_ | 252 |
|  | 1-Tetracosene | C_24_H_48_ | 336 |
|  | Squalene | C_30_H_50_ | 410 |
| Alcohols | (22E)-Ergosta-5,7,9(11),22-teeraen-3,β-ol | C_28_H_42_O | 394 |
|  | 5,6-dihydroergosterol | C_28_H_46_O | 398 |
|  | Eburicol | C_31_H_52_O | 440 |
|  | Lanosterol | C_30_H_50_O | 426 |
| Phenol | 24-methylene-lophenol | C_29_H_48_O | 412 |
| Acids | Linoleic acid | C_18_H_32_O_2_ | 280 |
|  | Oleic acid | C_18_H_34_O_2_ | 282 |
|  | Palmitic acid | C_16_H_32_O_2_ | 256 |
| Esters | Butyl phthalate | C_16_H_22_O_4_ | 278 |
|  | Dioctyl phthate | C_24_H_38_O_4_ | 390 |
|  | Ethyl linoleate | C_20_H_36_O_2_ | 308 |
|  | Ethyl oleate | C_20_H_38_O_2_ | 310 |
|  | Ethylene glycol oleate | C_20_H_38_O_3_ | 326 |
|  | Isobutyl phthalate | C_16_H_22_O_4_ | 278 |
|  | Isooctyl phthalate | C_24_H_38_O_4_ | 390 |
|  | Methyl avachate | C_21_H_42_O_2_ | 326 |
|  | Methyl behenate | C_23_H_46_O_2_ | 354 |
|  | Methyl cerotate | C_27_H_54_O_2_ | 410 |
|  | Methyl erucate | C_23_H_44_O_2_ | 352 |
|  | Methyl ester of 3-(3,5-di-tert-butyl-4- hydroxyphenyl) -propinonic acid | C_18_H_28_O_3_ | 292 |
|  | Methyl lignocerate | C_25_H_50_O_2_ | 382 |
|  | Methyl linoleate | C_19_H_34_O_2_ | 294 |
|  | Methyl margarate | C_18_H_36_O_2_ | 284 |
|  | Methyl octacosanoate | C_29_H_58_0_2_ | 438 |
|  | Methyl oleate | C_19_H_36_O_2_ | 296 |
|  | Methyl palmitate | C_17_H_34_O_2_ | 270 |
|  | Methyl palmitoleate | C_17_H_32_O_2_ | 268 |
|  | Methyl stearate | C_19_H_38_O_2_ | 298 |
|  | Methyl tricosanoate | C_24_H_48_O_2_ | 368 |
|  | Methyl-11-eicosenoate | C_21_H_40_O_2_ | 324 |
|  | palmityl palmitate | C_32_H_64_O2 | 480 |
| Nitrogenous compounds | 1,4-diaza-2,5-dioxo-3-isobutylbicyclo [4,3,0] nonane | C_11_H_18_N_2_O_2_ | 210 |
|  | 3-benzul-1,4-diaza-2,5-dioxobicyclo [4,3,0] nonane | C_14_H_16_N_2_O_2_ | 244 |
|  | Methyl N-(phenylacetyl) glycine | C_11_H_13_NO_3_ | 207 |
|  | Erucamide | C_22_H_43_NO | 337 |

Table 6 Compounds isolated from chloroform phase of *Alternaria oxytropis*

| Type | Compound | MF | MW |
| --- | --- | --- | --- |
| Alkanes | 1,1'-oxybis-dodecane | C_24_H_50_O | 354 |
|  | 2-methyl Tricosane | C_24_H_50_ | 338 |
|  | Docosane | C_22_H_46_ | 310 |
|  | 2-methyl-Hexadecane | C_17_H_36_ | 240 |
|  | 4-methyl-Heptadecane | C_18_H_38_ | 254 |
|  | Eicosane | C_20_H_42_ | 282 |
|  | Hentriacontane | C_31_H_64_ | 436 |
|  | Heptacosane | C_27_H_56_ | 380 |
|  | Heptadecane | C_17_H_36_ | 240 |
|  | Hexacosane | C_26_H_54_ | 366 |
|  | Hexadecane | C_16_H_34_ | 226 |
|  | Nonacosane | C_29_H_60_ | 408 |
|  | Nonadecane | C_19_H_40_ | 268 |
|  | Nor-pristane | C_18_H_38_ | 254 |
|  | Octacosane | C_28_H_58_ | 394 |
|  | Octadecane | C_18_H_38_ | 254 |
|  | Pentacosane | C_25_H_52_ | 352 |
|  | Phytane | C_20_H_42_ | 282 |
|  | Pristane | C_19_H_40_ | 268 |
|  | Triacontane | C_30_H_62_ | 422 |
|  | Tricosane | C_23_H_48_ | 324 |
|  | Tetracosane | C_24_H_50_ | 338 |
| Olefin | Squalene | C_30_H_50_ | 410 |
| Alcohols | 5-Acetyl-2-furanmethanol | C_7_H_8_O_3_ | 140 |
|  | 6-methyl-2-pyrazinylmethanol | C_6_H_8_N_2_O | 124 |
| Aldehydes | 4-(dimethylamino)-Benzaldehyde | C_9_H_11_NO | 149 |
|  | 5,5'-[oxobis (methylene)]bis-2-Furancarboxaldehyde | C_12_H_10_O_5_ | 234 |
|  | 5-Oxymethylfurfurole | C_6_H_6_O_3_ | 126 |
| Ketone | 2,3-dihydro-3,5-dihydroxy-6-methyl-4H-Pyran-4-one | C_6_H_8_O_4_ | 144 |
| Acids | 2-benzyl-3,6-dioxo-5-methylpiperazine | C_12_H_14_N_2_O_2_ | 218 |
|  | 2-benzyl-3,6-dioxopiperazine | C_11_H_12_N_2_O_2_ | 204 |
|  | 3-(m-aminobenzoyl)-2-methyl-Propionic acid | C_11_H_13_NO_3_ | 207 |
|  | 5-formyl-2-furfurylmethanoate | C_7_H_6_O_4_ | 154 |
|  | Benzeneacetic acid | C_8_H_8_O_2_ | 136 |
|  | Linoleic acid | C_18_H_32_O_2_ | 280 |
|  | Myristic acid | C_14_H_28_O_2_ | 228 |
|  | oleic acid | C_18_H_34_O_2_ | 282 |
| Esters | (+)-S-Pantolactone | C_6_H_10_O_3_ | 130 |
|  | Butyl phthalate | C_16_H_22_O_4_ | 278 |
|  | Dioctyl phthalate | C_24_H3_8_O_4_ | 390 |
|  | Lactone G | C_5_H_8_O_4_ | 132 |
|  | Ethyl 2-acetyl propionate | C_7_H_12_O_3_ | 144 |
|  | Ethyl oleate | C_20_H_38_O_2_ | 310 |
|  | Ethyl palmitate | C_18_H_36_O_2_ | 284 |
|  | Isobutyl phthalate | C_16_H_22_O_4_ | 278 |
|  | Isooctyl phthalate | C_24_H_38_O_4_ | 390 |
|  | Methyl behenate | C_23_H_46_O_2_ | 354 |
|  | Methyl ligocerate | C_25_H_50_O_2_ | 382 |
|  | Methyl linolelaidete | C_19_H_34_O_2_ | 294 |
|  | Methyl oleate | C_19_H_36_O_2_ | 296 |
|  | Methyl palmitate | C_17_H_34_O_2_ | 270 |
|  | Methyl palmitoleate | C_17_H_32_O_2_ | 268 |
|  | Methyl stearate | C_19_H_38_O_2_ | 298 |
|  | Methyl-4-hydroxyphenyl acetate | C_9_H_10_O_3_ | 166 |
|  | Monomethyl succinate | C_5_H_8_O_4_ | 132 |
|  | palmityl palmitate | C_32_H_64_O_2_ | 480 |
| Nitrogenous compounds | 1,4-diaza-2,5-dioxo-3-isobutyl bicyclo [4,3,0] nonane | C_11_H_18_N_2_O_2_ | 210 |
|  | 1,4-diaza-2,5-dioxobicyclo [4,3,0] nonane | C_7_H_10_N_2_O_2_ | 154 |
|  | 3-benzyl-1,4-diaza-2,5-dioxobicyclo [4,3,0] nonane | C_14_H_16_N_2_O_2_ | 244 |
|  | Benzeneacetamide | C_8_H_9_NO | 135 |
|  | Cycloalanylvaline | C_8_H_14_N_2_O_2_ | 170 |
|  | L-Phenylalanyl-L-proline lactam | C_14_H_16_N_2_O_2_ | 244 |
|  | Methyl N-(phenylacetyl) glycine | C_11_H_13_NO_3_ | 207 |
|  | Methyl pyroglutamate | C_6_H_9_NO_3_ | 143 |

Table 7 Compounds isolated from ethyl acetate phase of *Alternaria oxytropis*

| Type | Compound | MF | MW |
| --- | --- | --- | --- |
| Alkanes | Heptacosane | C_27_H_56_ | 380 |
|  | Nonacosane | C_29_H_60_ | 410 |
|  | Octacosane | C_28_H_58_ | 394 |
| Olefin | Squalene | C_30_H_50_ | 410 |
| Alcohols | 5,6-dihydroergosterol | C_28_H_46_O | 398 |
|  | Eburicol | C_31_H_52_O | 440 |
| Aldehydes | 4-(dimethylamino)-Benzaldehyde | C_9_H_11_NO | 149 |
|  | 5-Acetoxymethyl-2-furaldehyde | C_8_H_8_O_4_ | 168 |
|  | 5-Oxymethylfurfurole | C_6_H_6_O_3_ | 126 |
|  | Furfural | C_5_H_4_O_2_ | 96 |
| Acids | Benzeneacetic acid | C_8_H_8_O_2_ | 136 |
|  | Linoleic acid | C_18_H_32_O_2_ | 280 |
| Furan | 5-(Hydroxymethyl)-2-(dimethoxymethyl) furan | C_8_H_12_O_4_ | 172 |
| Ketone | 2,3-dihydro-3,5-dihydroxy-6-methyl-4H-Pyran-4-one | C_6_H_8_O_4_ | 144 |
|  | 3,5-dihydroxy-2-methyl-4H-Pyran-4-one | C_6_H_6_O_4_ | 142 |
| Esters | 4-hydrozyphenyllactic acid-methylester | C_10_H_12_O_4_ | 196 |
|  | Buryl phthalate | C_16_H_22_O_4_ | 278 |
|  | Dioctyl phthalate | C_24_H_38_O_4_ | 390 |
|  | Ethyl N-(O-anisyl)formimidate | C_10_H_13_NO_2_ | 179 |
|  | Ethyl phthalate | C_12_H_14_O_4_ | 222 |
|  | Isooctyl phthalate | C_24_H_38_O_4_ | 390 |
|  | L-methyl-3-phenyllactate | C_10_H_12_O_3_ | 180 |
|  | Methyl lignocerate | C_25_H_50_O_2_ | 382 |
|  | Methyl linoleate | C_19_H_34_O_2_ | 294 |
|  | Methyl N-(phenylacetyl) glycine | C_11_H_13_NO_3_ | 207 |
|  | Methyl oleate | C_19_H_36_O_2_ | 296 |
|  | Methyl palmitate | C_17_H_34_O_2_ | 270 |
|  | Methyl pyroglutamate | C_6_H_9_NO_3_ | 143 |
|  | Methyl stearate | C_19_H_38_O_2_ | 298 |
|  | Methyl tricosanoate | C_24_H_48_O_2_ | 368 |
|  | Monomethyl succinate | C_5_H_8_O_4_ | 132 |
|  | 1,4-diaza-2,5-dioxo-3-isobutyl bicyclo [4,3,0] nonane | C_11_H_18_N_2_O_2_ | 210 |
| Nitrogenous compounds | 1,4-diaza-2,5-dioxobicyclo [4,3,0] nonane | C_7_H_10_N_2_O_2_ | 154 |
|  | Erucamide | C_22_H_43_NO | 337 |
|  | Triacetonamine | C_9_H_17_NO | 155 |
