# Supplementary material for: The study of metabolites from fermentation culture of Alternaria oxytropis
Source: BMC Microbiol. 2019 Feb 11;19:35. doi: 10.1186/s12866-019-1408-8 (PMC6369557; doi:10.1186/s12866-019-1408-8)
Supplement: Supplementary file 2 — Figures S1-S16. The GC-MS and LC-MS analysis of petroleum ether, chloroform, ethyl acetate and n-butanol phase in metabolites of Alternaria oxytropis. (DOCX 1545 kb) [file 12866_2019_1408_MOESM2_ESM.docx]

Fig. S_1_ The GC-MS total ion stream chromatogram of S-1 segment of petroleum ether of *Alternaria oxytropis*

Fig. S_2_ The GC-MS total ion stream chromatogram of S-2 segment of petroleum ether of *Alternaria oxytropis*

Fig. S_3_ The GC-MS total ion stream chromatogram of S-3 segment of petroleum ether of *Alternaria oxytropis*

Fig. S_4_ The GC-MS total ion stream chromatogram of L-1 segment of chloroform phase of *Alternaria oxytropis*

Fig. S_5_ The GC-MS total ion stream chromatogram of L-2 segment of chloroform phase of *Alternaria oxytropis*

Fig. S_6_ The GC-MS total ion stream chromatogram of L-3 segment of chloroform phase of *Alternaria oxytropis*

Fig. S_7_ The GC-MS total ion stream chromatogram of Y-1 segment of ethyl acetate of *Alternaria oxytropis*

Fig.S_8_ The GC-MS total ion stream chromatogram of Y-2 segment of ethyl acetate of *Alternaria oxytropis*

Fig. S_9_ The GC-MS total ion stream chromatogram of Y-3 segment of ethyl acetate of *Alternaria oxytropis*

Fig. S_10_ Mass spectra of typical composition of JZ1 column chromatography from *Alternaria oxytropis* fermentation broth

Fig. S_11_ Mass spectra of typical composition of JZ2 column chromatography from *Alternaria oxytropis* fermentation broth

Fig. S_12_ Mass spectra of typical composition of JZ3 column chromatography from *Alternaria oxytropis* fermentation broth

Fig. S_13_ Mass spectra of typical composition of JZ4 column chromatography from *Alternaria oxytropis* fermentation broth

Fig. S_14_ Mass spectra of typical composition of JZ5 column chromatography after acetylated from *Alternaria oxytropis* fermentation broth(A)

Fig.S_15_ Mass spectra of typical composition of JZ5 column chromatography after acetylated from *Alternaria oxytropis* fermentation broth(B)

Fig. S_16_ Mass spectra of typical composition of JZ5 column chromatography after acetylated from *Alternaria oxytropis* fermentation broth(C)
